# Measuring the Thermal Unfolding of Lysozyme: A Critical Comparison of Differential Scanning Fluorimetry and Differential Scanning Calorimetry

**DOI:** 10.1002/open.202400340

**Published:** 2025-02-11

**Authors:** Weronika Malicka, Yueyue Dai, Andreas Herrmann, Rainer Haag, Matthias Ballauff, Marina Pigaleva, Thomas Risse, Daniel Lauster, Iman Asakereh, Mazdak Khajehpour

**Affiliations:** ^1^ Institute of Chemistry and Biochemistry Freie Universität Berlin 14195 Berlin Germany; ^2^ Institut für Pharmazie Freie Universität Berlin 14195 Berlin Germany; ^3^ University of Manitoba Winnipeg Manitoba R3T 2 N2 Canada

## Abstract

The thermal unfolding of lysozyme in aqueous solution has been analyzed by (nano) differential scanning fluorimetry (nanoDSF) and differential scanning calorimetry (DSC). In addition, dynamic light scattering (DLS) acquired in parallel to the DSF measurements, was used to confirm that the change in hydrodynamic radius upon unfolding is rather small (*R*_*H,f*_ =1.75 nm in the folded state; and *R*_*H,u*_=1.91 nm in the unfolded state). NanoDSF measurements were evaluated to characterize the folding/unfolding transition within the classical two‐state folding model. The temperature of unfolding (*T_m_*) is found to be the most robust quantity. The unfolding enthalpy $\Delta H_{u}$ and the change of specific heat were also obtained and errors in the range of 5–10 % and 30–50 % were determined, respectively. A comparison of thermodynamic parameters from nanoDSF and DSC measurements provides evidence for an increasing unfolding enthalpy $\Delta H_{u}$ with protein concentration. A comparison with data from literature suggests that a weak association in the folded state can lead to the observed change of the unfolding enthalpy. For *Δc_p_* significantly higher values is deduced from the analysis of temperature dependent nanoDSF measurements (10 kJ/(K mol)) as compare to DSC (3–5 kJ/(K mol)).

## Graphical abstract

The thermal unfolding of lysozyme in aqueous solution is analyzed by (nano) differential scanning fluorimetry (nanoDSF) and differential scanning calorimetry (DSC). Dynamic light scattering (DLS) in parallel to the DSF measurements shows the expansion of the protein during unfolding. The combination of DSF, DSC, and DLS leads to a complete and detailed picture of the unfolding transition.

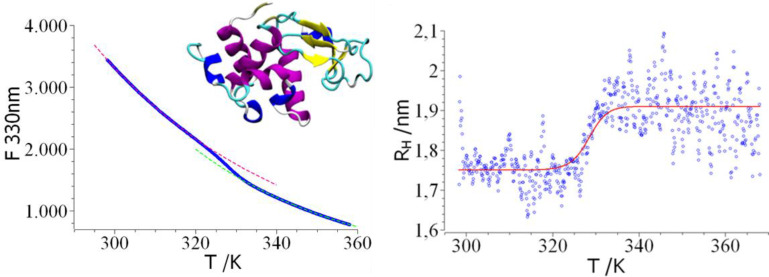

## Introduction

The thermal stability of proteins is a long‐standing problem of biophysics.^1^ Heated to a well‐defined temperature *T_m_* will result in a melting of the spatial structure of the protein into a unfolded state. For a number of proteins, denaturation can be treated in terms of a first order transition where the folded and the unfolded state are in equilibrium at each point.[^2^, ^3^, ^4^] Thus, a thermodynamic discussion of the various factors influencing the thermal stability can be done. In particular, Hofmeister effects introduced by salts in aqueous solution will lead to marked changes of T_m_.[^4^, ^5^, ^6^, ^7^, ^8^, ^9^, ^10^, ^11^, ^12^, ^13^, ^14^, ^15^, ^16^] In this way, the unfolding transition of proteins becomes a well‐defined thermodynamic problem that can be studied directly by methods as differential scanning calorimetry (DSC).[^3^, ^7^, ^17^, ^18^, ^19^] Thus, careful studies by DSC have been used to unravel Hofmeister‐effects on various proteins.[^12^, ^20^, ^21^, ^22^, ^23^] Measurements by DSC, however, necessitate considerable amounts and rather high concentrations of the protein. In contrast to that structural changes on the level of the backbone fold can be obtained at lower concentrations using spectroscopic methods such as CD‐spectroscopy,[^24^, ^25^, ^26^, ^27^] UV‐spectroscopy[^28^, ^29^] or fluorescence spectroscopy.[^30^, ^31^] In case the unfolding transition is a well‐defined two‐step process, both DSC and spectroscopic methods should yield the same thermodynamic information.^[32]^ However, differences in the experimental conditions can alter these properties which renders comparative studies on a well‐controlled model protein highly useful to assess the accuracy of the thermodynamic parameters obtained from both methods.

Fluorescence spectroscopy is certainly the most sensitive and thus preferable method as it allows us to work at the lowest protein concentration possible. Unfolding of the protein will change the fluorescence of the aromatic side chains ‐dominated by the fluorescence of tryptophan and tyrosine residues‐ which leads to a red‐shift in emission wavelength as well as a change in fluorescence intensity. In differential scanning fluorimetry (DSF)[^31^, ^33^, ^34^, ^35^] the fluorescence intensity of a dissolved protein is typically measured at two different wavelengths as the function of temperature and subsequently analyzed to give the degree of unfolding *α*. as a function of temperature. Within a two‐state folding model a thermodynamic analysis of *α(T)* can yield in principle 3 different parameters, namely the transition temperature *T_m_*, the transition enthalpy *ΔH_u_*, and the change *Δc_p_* of the specific heat. Additional justification for the two‐state folding model can be obtained from cooling experiments in case the transition is found to be reversible. Often the change of intrinsic fluorescence occurring upon unfolding is not large enough. Therefore, addition of solvatochromic dyes[^36^, ^37^, ^38^, ^39^] or tagging by the green fluorescent protein^[40]^ was used to enhance the effect upon unfolding. In this respect, *SYPRO Orange* has been frequently used as a dye since it binds to hydrophobic patches of the protein that become exposed during unfolding.[^36^, ^37^, ^38^, ^39^, ^41^, ^42^] However, additives such as dyes can change the transition markedly as e. g. shown for the case of lysozyme by Liu and coworkers.^[43]^

In many studies, the analysis by DSF is restricted to monitoring *T_m_* as the function of a co‐solute or to check the purity of a given protein.[^34^, ^35^, ^36^, ^38^] The determination of the transition enthalpy *ΔH_u_*, and the change *Δc_p_* of the specific heat, on the other hand, requires high quality data as well as an appropriate analysis to derive the degree of unfolding *α* as the function of temperature with sufficient accuracy.[^41^, ^42^] The change of specific heat (*Δc_p_*) has turned out to be a quantity which is particularly difficult to obtain, even if highly precise DSC‐experiments are used (cf. the discussion by Pace and coworkers^[44]^). Hence, in many evaluations of DSF‐experiments *Δc_p_* is simply omitted. Here the question arises whether *Δc_p_* which is a central thermodynamic parameter can be determined by DSF with sufficient accuracy.

In this study we employ a nanoDSF device (Prometheus Panta PNT‐00203, Nanotemper technologies Germany) to monitor the olding/unfolging transition of lysozyme exploiting its natural fluorescence for concentrations down to the micromolar range and volumes of only 20 μL. Hence, investigations on proteins available only in very small quantities become possible. Even though thermodynamic properties are automatically extracted from the nanoDSF measurement, significant variations were observed which prompted us to look in detail into the analysis procedure and determine the error bars associated with the different thermodynamic quantities. To assess the reliability of these values, studies on a model system and comparison to other well‐established techniques are required. To this end, lysozyme is a perfect choice as it offers the opportunity to compare to DSC measurements (also reported in literature[^1^, ^17^, ^42^, ^45^, ^46^, ^47^, ^48^, ^49^, ^50^, ^51^, ^52^]) and reports from other spectroscopic methods such as UV‐Vis‐spectroscopy[^53^, ^54^] allowing for a detailed discussion of the results obtained here.

## Theory and Evaluation of Data

### Thermodynamics of the Two‐State Folding Model

Within this model, the folded state is assumed to be in equilibrium with the unfolded state at each temperature. The equilibrium constant *K_u_* defined in this way yields the standard value of the free enthalpy $\Delta G_{u}^{0}$ of unfolding[^4^, ^32^, ^55^]

(1) $$\Delta G_{u}^{0}=\;-RTln\;K_{u}$$

$\Delta G_{u}^{0}$ depends on temperature by

(2) $$\Delta G_{u}^{0}=\Delta H_{u}^{0}\left(1-\;\frac{T}{T_{m}}\right)+\;\Delta c_{p}\left(T-\;T_{m}-Tln\frac{T}{T_{m}}\right)$$

where $\Delta H_{u}^{0}$ denotes the enthalpy of unfolding whereas $\Delta c_{p}$ denotes the change of specific heat during the transition taking place at $T_{m}$ . The degree of unfolding *α* i. e. the molar fraction of unfolded protein is related to the equilibrium constant through

(3) $$\alpha =\;\frac{K_{u}}{1+K_{u}}$$

and can be expressed by

(4) $$\alpha =\;\frac{exp(-\frac{\Delta G_{u}^{0}}{RT})}{1+exp(-\frac{\Delta G_{u}^{0}}{RT})}$$

where $\Delta G_{u}^{0}$ is given by eq.(2). The question arises whether the accuracy of the degree of unfolding is good enough to allows the determination not only of $\Delta H_{u}^{0}$ but also of $\Delta c_{p}$ . In the following we will for brevity refer to the thermodynamic properties as ${\Delta G}_{u}$ and ${\Delta H}_{u}$ .

Given the validity of the two‐state folding model, the experimental data can be evaluated in terms of the degree of unfolding α. We shall first discuss the evaluation of the data obtained by DSF and subsequently the information obtained from DSC. Special emphasis is put on the determination of *Δc_p_* which presents a central piece of thermodynamic information.

### Evaluation of DSF‐Data

The evaluation of the degree of unfolding *α* from fluorescence spectroscopy was recently discussed in detail by Zoldak et al.^[56]^ In principle, *α* could be obtained from the ratio of the intensities measured at 350 nm and at 330 nm. The rationale behind this method is the observation that the strong dependence of the intensities on temperature (see below) can be mostly removed in this way. However, Zoldak et al. demonstrated that a determination of *α* solely from the ratio of the intensities measured at 350 nm and 330 nm can lead to considerably errors.[^56^, ^57^] Hence, we adopted an interactive procedure which allows for an assessment of the different steps in the analysis:

Within the two‐state folding model, the measured intensity *F_350_* and *F_330_* can be split into^[57]^

(5) $$F_{350}=\;\frac{F_{f,350}+F_{u,350}K_{u}}{1+K_{u}}$$

(6) $$F_{330}=\;\frac{F_{f,330}+F_{u,330}K_{u}}{1+K_{u}}$$

where *F*_*f,350*_ and *F*_*u,350*_ denote the intensities of fluorescence at a wavelength of 350 nm in the folded and the unfolded state, respectively, whereas *F*_*f,330*_ and *F*_*u,330*_ refer to the respective quantities measured at a wavelength of 330 nm. Hence, four different intensities *F*_*f,350*_ *, F*_*u,350*_ *, F*_*f,330*_, and *F*_*u,330*_ must be determined. These intensities have to be obtained from fits of the measured intensities *F_350_* and *F_330_*. Within the two‐state folding model the system will be in the folded and the unfolded state well below or above the transition temperature, respectively. Hence, the temperature dependence of the fluorescence in each state which oftentimes exhibits a pronounced non‐linear behavior as discussed extensively by Eftink^[30]^ can be determined if the curves have been determined for a sufficiently wide temperature ranges below and above the transition temperature. In case a small range of temperatures is considered, this dependence may be approximated by a linear function.^[30]^ However, the intensities *F_f_(T)* in the folded state and of *F_u_(T)* of the unfolded state must be extrapolated over a comparably wide range of the temperature below and above the transition. Therefore, the extrapolation of the fluorescence of the two states into the region in which their concentration is minute becomes a critical factor for the precision of the subsequent analysis. Here we found that in many cases an exponential provides an accurate description of the observed intensities:

(7) $$F_{f}\left(T\right)=a^{*}exp\left(-b^{*}T\right)$$

In some cases, however, a linear fit proves to be a better description of the data which stresses the necessity for an interactive procedure in which the quality of each step is critically assessed. In all cases an excellent fit of *F_f_(T)* and *F_u_(T)* in the entire one‐phase regions, respectively, is key for an accurate determination of thermodynamic quantities as this is a mandatory requirement for a meaningful extrapolation.

With a good fit of *F_f_(T)* and *F_u_(T)*, the degree of unfolding *α* can be determined from the data obtained at a single wavelength e. g. 350 nm by:

(8) $$\alpha =\;\frac{F_{350}-\;F_{f,350}}{F_{u,350}-\;F_{f,350}\;}$$

In this way the unfolding can be monitored for the two available wavelength and compared to each other. In addition, from eq.(1) and (2) it follows that the ratio *R* of the two measured intensity is given by

(9) $$\frac{F_{350}}{F_{330}}=R=\;\frac{F_{f,350}+F_{u,350}K_{u}}{F_{f,330}+F_{u,330}K_{u}}$$

Hence,

(10) $$K_{u}=\;\frac{F_{f,350}-RF_{f,330}}{RF_{u,330}-F_{u,350}}$$

from which the degree of unfolding can be calculated through eq.(3). Application of eq.(10) has the advantage that data taken at two different wavelengths are evaluated together which minimizes possible statistical errors of the data. On the other hand, solutes like salts or sucrose may shift the fluorescence spectra considerably so that the intensities at 330 nm and at 350 nm refer to different states. In this case, the evaluation must proceed through eq.(8).

As an alternative, a global fit can be done by fitting *α* to the entire intensity *F_350_*. Here we combine

(11) $$F_{350}=\left(1-\alpha \right)F_{f,350}+\alpha F_{u,350}$$

with eq.(4), (2) and (7) so that all parameters including *a* and *b* in eq.(7) are fitted in one global, non‐linear least square fit using e. g. a Levenberg‐Marquard algorithm (the analysis of the data shown here were done using its implementation in Matlab). It should be kept in mind that this fit has four additional adjustable parameters, namely the parameters *a* and *b* for the folded and the unfolded regime, respectively, which may allow for a compensation of errors. A comparison of both ways of evaluation may hence give direct information about possible errors incurred for the various thermodynamic parameters.

### Evaluation of DSC‐Data

Here we follow mostly the established way of evaluation specified in two important points.[^1^, ^47^, ^48^, ^58^, ^59^] The normalized heat signal *C_p_* can be approximated linearly in the folded state by

(12) $$C_{p,f}=a_{f}+b_{f}T$$

For the unfolded state we have similarly

(13) $$C_{p,u}=a_{u}+b_{u}T$$

With these definitions, the background can be obtained as follows

(14) $$C_{bg}=C_{p,f}\left(1-\alpha \right)+C_{p,u}\alpha$$

The entire *C_p_(T)* now follows as

(15) $$C_{p}\left(T\right)=\;C_{bg}+C_{p}^{ex}$$

The quantity $C_{p}^{ex}$ defines the net caloric effect of protein unfolding.

From general thermodynamics we have

(16) $$\frac{dlnK_{u}}{dT}=\;\frac{\Delta H_{vH}}{RT^{2}}=\;\frac{\Delta H_{u}}{RT^{2}}$$

Please note that the enthalpy of the process usually termed van't Hoff enthalpy (*ΔH_vH_)* are identical to the enthalpy of transition *ΔH_u_* if the two‐state folding model is adopted. Hence, in a self‐consistent treatment of protein denaturation, both quantities must agree within prescribed limits of error. With eq.(1) we have^1^

(17) $$\frac{dlnK_{u}}{dT}=\;\frac{\Delta H_{u}\left(T\right)}{RT^{2}}=\;\frac{1}{\alpha (1-\alpha )}\;\frac{d\alpha }{dT}$$

In the course of an analysis of DSC‐data, the necessary assumption is that

(18) $$\frac{d\alpha }{dT}=\;\frac{C_{p}^{ex}}{\Delta H_{cal}}$$

where *ΔH_cal_* is the measured caloric enthalpy of transition derived from experimental data through

(19) $$\Delta H_{cal}=\;\int C_{p}^{ex}dT$$

There is no reason whatsoever that *ΔH_cal_* should agree in all cases with *ΔH_u_*. The caloric enthalpy *ΔH_cal_* contains all effects as e.g the heat of ionization of the buffer whereas *ΔH_vH_* =*ΔH_u_* is precisely defined by eq.(16). Taking together eq.(17) and (18) and expressing the temperature dependence of $\Delta H_{u}\left(T\right)$ by its value at the melting temperature ($T_{m}$ ) and the change in heat capacity associated with the process ($\Delta c_{p}$ ), we obtain for $C_{p}^{ex}$ the following expression:

(20) $$C_{p}^{ex}\left(T\right)=\frac{\left[\Delta H_{u}\left(T_{m}\right)+\Delta c_{p}\left(T-T_{m}\right)\right]\Delta H_{cal}}{RT^{2}}\alpha \left(1-\alpha \right)$$

Evidently, $C_{p}^{ex}$ is related to the degree of unfolding *α* by two well‐defined and different enthalpies. Note that $\Delta H_{cal}$ is a constant while $\Delta H_{u}$ depends on temperature.

Another way of calculating $\Delta H_{u}\left(T_{m}\right)$ follows directly from eq.(17): For *T*=*T_m_* and α=0.5 and we get^[7]^

(21) $$H_{u}\left(T_{m}\right)=4RT_{m}^{2}\frac{C_{p}^{ex}\left(T_{m}\right)}{\Delta H_{cal}}$$

Evidently, both ways eq.(20) and eq.(21) must come to the same value of *ΔH_u_*.

### Evaluation of the Hydrodynamic Radius

Within the frame of the two‐step folding model, the overall dimensions of a protein must be characterized by exactly two hydrodynamic radii, namely the hydrodynamic radius $R_{H,f}$ in the folded state and $R_{H,u}$ characterizing the unfolded state. Since the two states are always at equilibrium during the transition, the actually measured hydrodynamic radius *R_H_* must be a linear superposition of both radii weighed by the degree of unfolding:

(22) $$R_{H}=\;R_{H,f}\left(1-\alpha \right)+R_{H,u}\alpha$$

A comparison of the measured *R_H_* with eq.(23) provides another way of checking the assumption of a two‐state folding model.

## Experimental

Hen egg‐white lysozyme was purchased from Sigma (CAS‐Number: 12650–88‐3) and used without further purification. Solutions with a protein concentration typically 10 μM were prepared in a glycine buffer (pH 2.0 and 2.8) or citrate buffer (pH 4–6). Both buffers had a concentration of 50 mM.

### nanoDSF

All measurements have been done using a nanoDSF device Prometheus Panta PNT‐00203 (Nanotemper technologies, Germany). For these measurements capillaries were typically filled with 20 μL protein solution with the typical concentration of 10 μM. The measurement of the unfolding and refolding of the protein has been done by heating and cooling between 25 °C and 77 °C using a rate of 0.5 °C per minute. This resulted in 18.18 points/°C. The signal was obtained by setting the power level of the excitation laser (280 nm) to 100 %. The measurements were always done at least with double repetitions.

### DSC

DSC experiments were performed using a Nano DSC (TA Instruments) with 0.3 mL capillary cell volume. Samples were equilibrated at 20 °C and then heated to 85 °C with a scan rate of 1 °C per minute and at 3 atm. Samples were degassed for 10 min before loading cells and pressurized at 3 atm to avoid bubble formation.[^12^, ^23^]

## Results and Discussion

### Analysis of the nanoDSF‐Data

**Figure 1:**
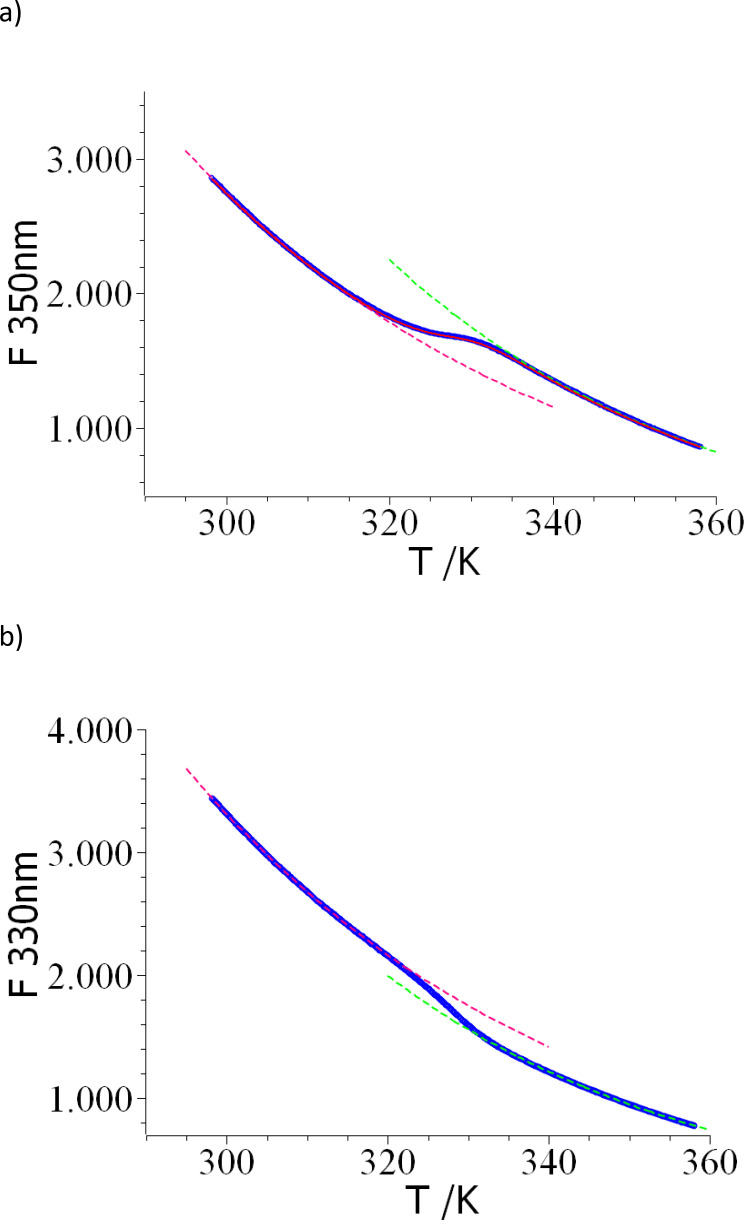
a,b. Thermal unfolding of lysozyme (concentration: 10 μM in a 50 mM glycine buffer) measured by nanoDSF at pH 2 as the function of temperature. monitored by DSF. 1a): The intensity of fluorescence excited at 280 nm measured at a wavelength 350 nm (blue circles) and at 330 nm in 1b). The dashed lines display the fits to the folded state (red dashed line) and the unfolded state (green dashed line) according to eq.(7), respectively, in both cases. The red solid line in Figure 1a shows the global fit according to eq.(11). See text for further explanation.

**Figure 2:**
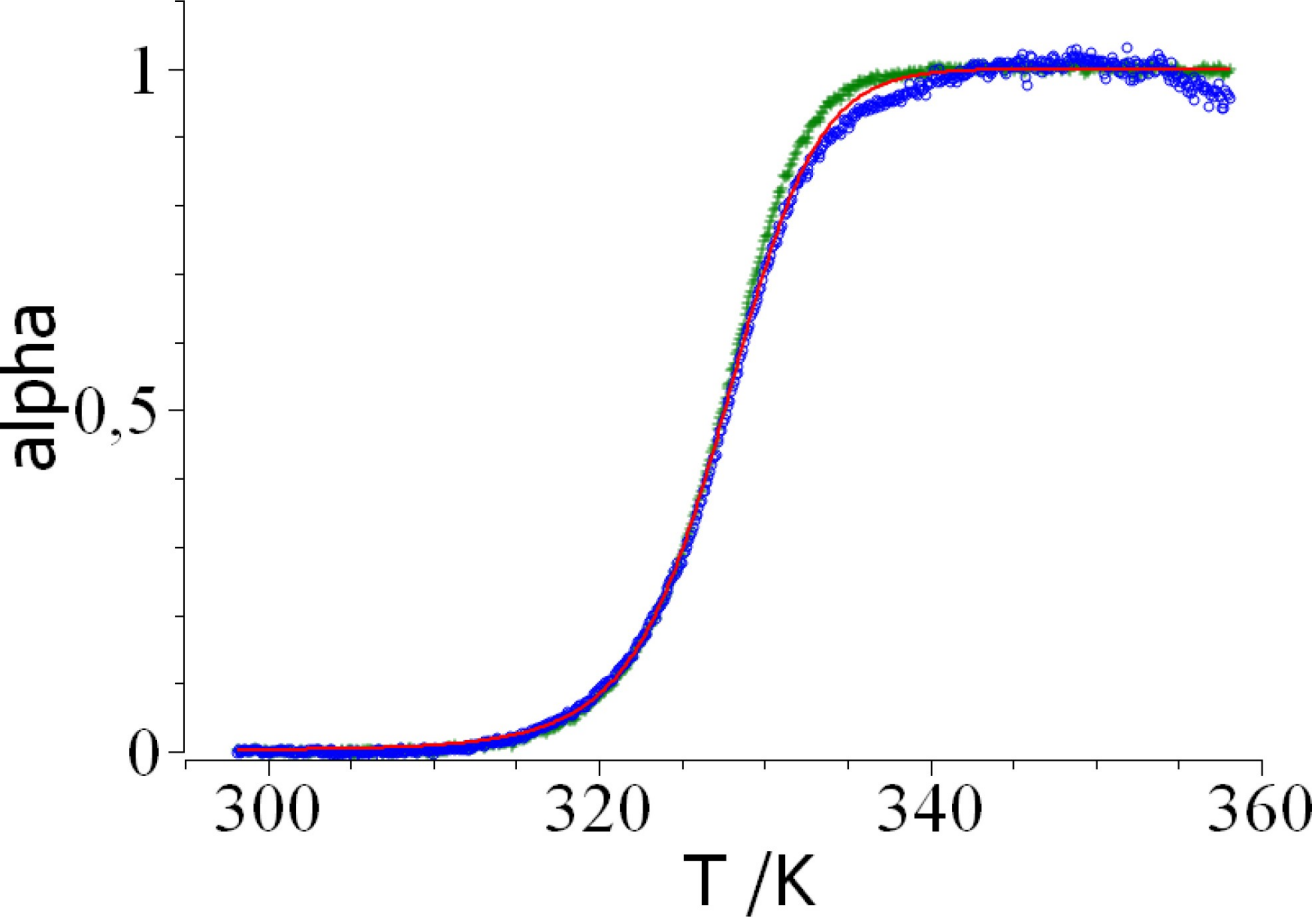
Degree of unfolding as calculated from the data solely taken at 350 nm (blue circles; see Figure 1a and from data taken at 330 nm and 350 nm by eq.(8)) and by eq.(10) and (3) (green crosses). The red solid line displays the fit of eq.(4) and eq.(2) to the experimental data.

We first discuss the unfolding of lysozyme in buffer solution at pH 2. Figure 2 displays the intensities of the intrinsic fluorescence measured at 330 nm and at 350 nm as a function of temperature. It should be kept in mind that no fluorescent dye has been added. The accuracy and reproducibility of the intensities is very good despite the fact that all data have been obtained at a protein concentration of just 10 μM. The change of fluorescence upon unfolding is more pronounced for the data obtained at 350 nm as expected for the fluorescence of tryptophan. In principle, the transition temperature *T_m_* could be estimated directly from the point of inflection of the curve in Figure 1a. Alternatively, the ratio *F_350_/F_330_* can be plotted against temperature and evaluated. As mentioned above, this procedure may lead to considerable errors as demonstrated by Zoldak and coworkers.^[56]^ Therefore we fit the intensity of fluorescence as the function of temperature below and above the transition point by eq.(7) allowing to extrapolate the intensities *F_f_(T)* and *F_u_(T)* into the two‐phase region. Evidently, the dependence of both *F_f_(T)* and *F_u_(T)* on *T* is non‐linear and linear fits would lead to erroneous results. The dashed lines in Figure 1a and b show the exponential fits (eq.(7)) of the intensities below and above the transition. The fits lead to an accurate determination of *F_f_(T)* and *F_u_(T)* so that both functions can be securely extrapolated into the temperature region in which the transition takes place.

For the evaluation of the degree of unfolding *α* one may use either eq.(8) or eq.(10) in conjunction with eq.(3). As already discussed above, an evaluation of both sets of data obtained at 350 nm and 330 nm at the same time would be preferable over evaluating the fluorescence data obtained at a single wavelength only. Figure 2 displays the respective data. Both sets agree except for the region in which *α*>0.95. Here an analysis of *K_u_* as determined by eq.(10) shows that this constant exhibits an appreciable error in this region and may become even negative. This observation points to problems of the evaluation using the results of both wavelength whereas no numerical problems are seen when evaluating the data through eq.(8). Moreover, these difficulties are even aggravated in presence of solutes as NaCl or sucrose which may lead to a considerable shift of the fluorescence spectra. Hence, the data obtained from a single wavelength through eq.(8) are more reliable and used in all subsequent experiments.

In the next step eq.(4) together with eq.(2) can be used for a thermodynamic evaluation of the degree of unfolding *α*. This fit is done using the MatLab routine *cftool* and leads to the unfolding temperature *T_m_*, the enthalpy of unfolding $\Delta H_{u}$ and the change of the specific heat $\Delta c_{p}$ . The red solid line in Figure 2 marks the resulting theoretical *α* derived from the fit. Full agreement of the fit with the experimental data is seen over the entire range of temperatures. This observation indicates that the two‐step folding model as expressed through eq.(2)–(4) gives a good description of the experimental data at pH 2 as expected.

**Figure 3:**
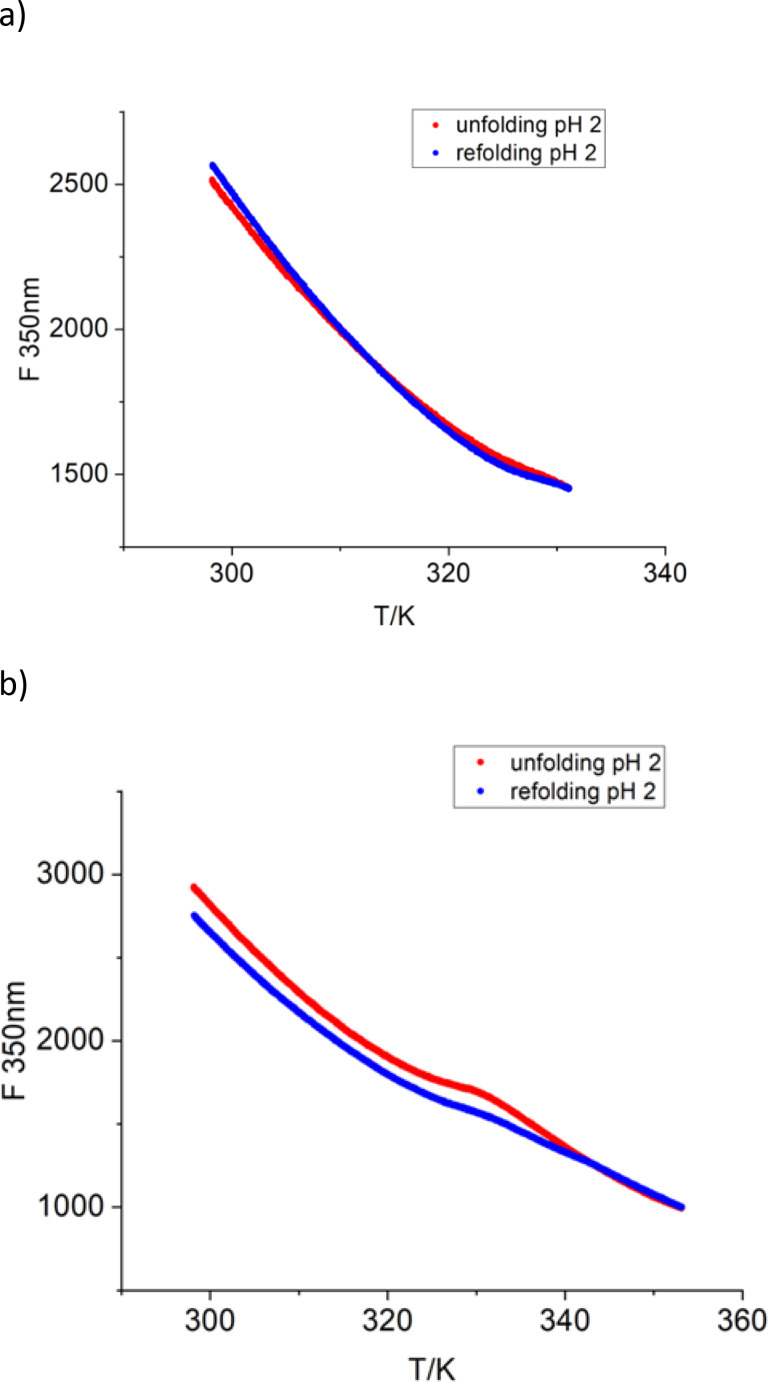
a,b Refolding experiments done at pH 2.0. Unfolding (blue) and refolding (red) of 10 μM lysozyme measured at 350 nm. a) in the temperature range of 298–331 K. (b) within the temperature range of 298–353 K.

It should be noted that the determination of *F_f_(T)* and *F_u_(T)* is done independently of this thermodynamic analysis. Hence, the accuracy of the description of both partial intensities can be assessed in each point. As an alternative, the fit of *F_f_(T)* and *F_u_(T)* and the analysis of *α* could be done in one step by use of eq.(11). Such a procedure has been applied frequently to experimental data obtained by UV/VIS or fluorescence.^41^ However, this evaluation may mask inconsistencies in the fits of *F_f_(T)* and *F_u_(T)* and the resulting parameters should be compared in all cases with the results of the step‐wise fit. Figure 1a displays such a global fit to the data shown in Figure 1a. Good agreement is found and a quantitative description is possible for all sets of data under consideration here. From the fit of the degree of unfolding *α* (see red solid line in Figure 1a) we obtain *T_m_* =327.6 K, $\Delta H_{u}$ =313 kJ/mol, and *Δc_p_* =10 kJ/(K mol). From the global fit (cf. Figure 3) we get *T_m_* =327.5, $\Delta H_{u}$ =307 kJ/mol, and *Δc_p_* =13 kJ/(K mol). The differences between the thermodynamic results from both methods of evaluation can give a good assessment of the limits of error of the thermodynamic parameters: In general, the transition temperature *T_m_* can be determined with excellent accuracy (±0.2 K). The temperature of the unfolding transition is hence the most secure parameter to be obtained from the experiment. The enthalpy of transition $\Delta H_{u}$ is afflicted by a considerably larger error of the order of ±15 kJ/mol (5–10 %) which needs to be discussed for each set of data (see below). The change of specific heat *Δc_p_*, however, is afflicted by an error of more than 30 %.

### Reversibility

The foregoing thermodynamic analysis requires reversibility in the range of temperatures in which *α* is raising from 0 to 1. This important point can be checked conveniently by monitoring the intensity of fluorescence during a cooling run. Figure 3a,b display two typical examples for this analysis. Figure 3a shows the signal measured during heating in blue and the respective signal upon cooling (red) when the sample was heated to just above the transition only. Full reversibility is seen. Figure 3b, on the other hand, shows the runs if the sample was heated to a higher temperature above the transition point. Here small changes are seen but the fluorescence signal is largely recovered. If the sample is heated to 363 K, however, no reversibility is seen and the signal of the cooling curve differs strongly from the signal of the heating curve (data not shown). Heating of the protein solution to temperatures near the boiling point of water obviously destroys a part of the lysozyme structure by e. g. partial hydrolysis so that no refolding results upon cooling. A similar analysis done for pH 2.8, 4, 5, and 6 is shown in Figures S1–S4 of the supplementary information. It demonstrates that the transition is reversible except for pH 6. This finding is in full agreement with literature as shown in the extensive discussion by Eftink^[30]^ and by Blumlein and McManus.^[60]^

### Dependence on Protein Concentration in DSF

The obvious advantage of nanoDSF as measured by the Panta‐device is ability to investigate proteins at low concentrations as compared to the protein concentrations necessary for a DSC‐experiment. While 10 μM protein concentration is sufficient to obtain high quality data, it is important to analyze to which extent the resulting thermodynamic parameters are depending on this parameter. To this end, we have performed nanoDSF measurements at different concentrations and comparative DSC experiments to elucidate the comparability of the two methods in more detail.

**Figure 4:**
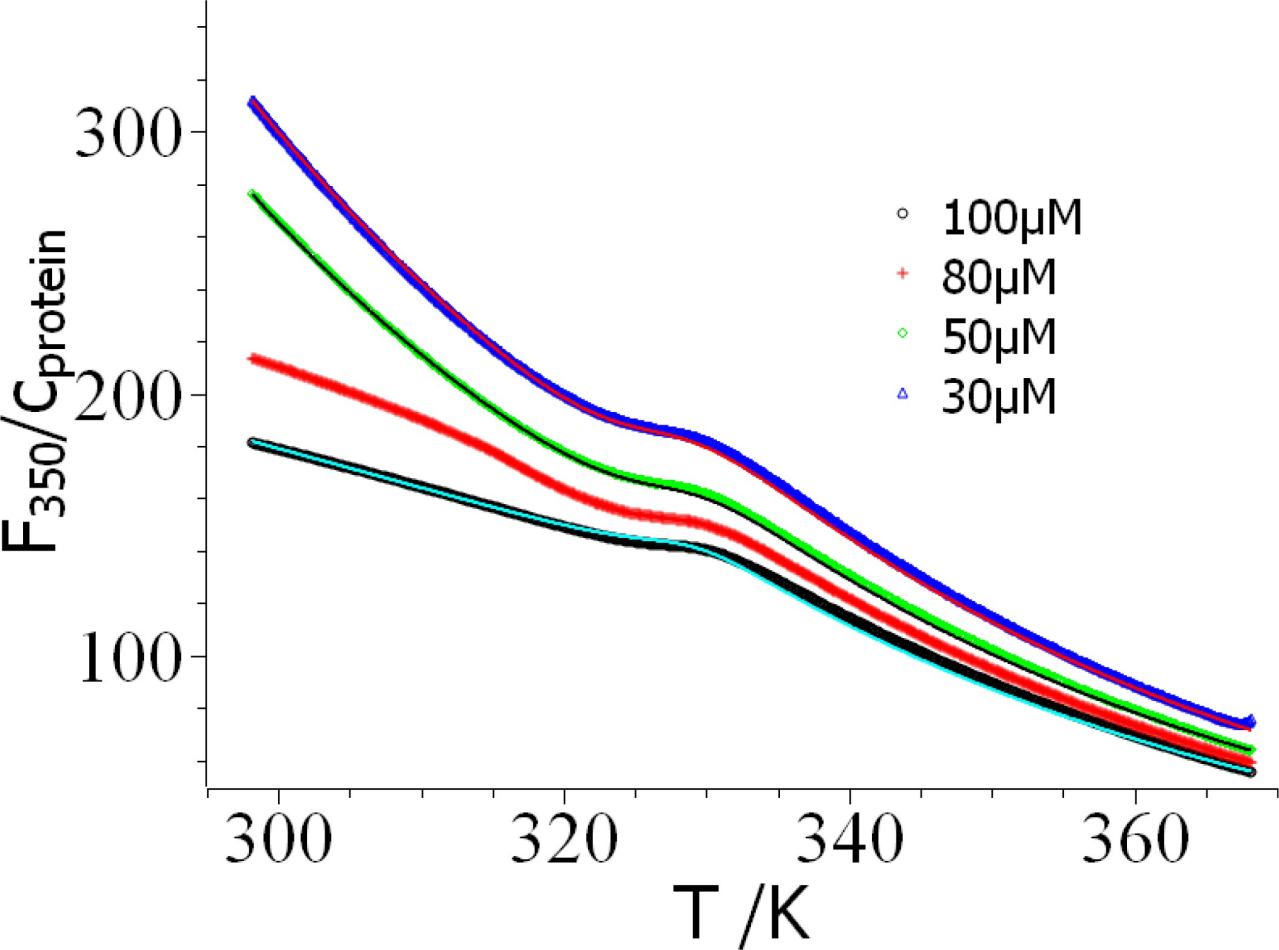
Analysis of the dependence of thermodynamic parameter on protein concentration. The intensity of fluorescence measured at 350 nm divided by the concentration of lysozyme is plotted against the temperature. The solid lines display the global fits of the respective data. No fit was possible for the data measured at a protein concentration of 80 μM. The respective fit parameters are gathered in table 1.

In principle, *F_350_*/c_protein_ measured for different concentrations of protein should overlap if there is no influence of this parameter. The plot of *F_350_/c_protein_* vs. *T* shown in Figure 4, however, demonstrates that there is a strong influence of *c_protein_* on the measured fluorescence below the unfolding transition. However, the data taken above *T_m_* virtually agree. While it is possible to describe the temperature dependent decay of the fluorescence at 350 nm (*F_350_*) by an exponential for small protein concentrations significant deviations are observed at 80 μM leading to an almost a linear dependence for *c_protein_* =100 μM below the transition. This finding already demonstrates that raising the concentration of lysozyme leads to profound changes in the interaction of the protein.

The data was analyzed by fitting *F_350_* below and above the transition using exponentials as described above for all but the curve for *c_protein_* =100 μM. Please note that the data obtained at 80 μM exhibits a clear change in curvature around 316 K which renders a reasonable fitting of this data set within the given model impossible. The data obtained for *c_protein_* =100 μM cannot be described using an exponential decay, instead *F*_*f,350*_ was fitted using a linear approximation. Table 1 gather the respective thermodynamic parameters. The strong dependence of *F_350_* below the transition is reflected in a marked increase of $\Delta H_{u}$ with protein concentration.

**Table 1:** Thermodynamic parameters for the unfolding of lysozyme.

Method	*c_p_* /μM	pH	*T_m_* /K	*ΔH_u_* /kJ/mol	*Δc_p_* /kJ/(K mol)	*ΔH_cal_* /kJ/mol	*ΔH_u_/ΔH_cal_*
DSF	10	2	327.6± 0.2	313± 20	10± 5	–	–
DSF	30	2	327.00.2±	339± 16	11±4	–	–
DSF	50	2	327.0±0.2	339±20	(14±6)	–	–
DSF	100	2	328.4±0.2	424±15	(10.2±4)	–	–
DSF	10	2.8	339.7± 0.4	393± 21	14.22.2±	–	–
DSF	10	4	348.3± 0.5	463± 57	15.83.3±	–	–
DSF	10	5	348.10.1±	467± 18	15.91.5±	–	–
DSF	10	6	345.60.3±	420± 27	9.8± 14.7	–	–
DSC	69	2	325.7±0.2	375 ±10	2.78±0.5	344.8±5	1.09
DSC	173	2	325.7±0.2	400±10	4.46±0.5	304.3±5	1.31

Since the protein concentrations are rather high, the question arises whether the measurements of the intensity are disturbed by an inner filter effect.^[61]^ However, the extinction coefficient of lysozyme is ~36000 which at a concentration of 100 micromolar for a capillary tube having an ID of 0.5 mm translates into an optical density of less than 0.2 at the highest concentrations studied. This leads to a small contribution of the inner filter effect at the concentrations studied. Also, because the optical density of the folded and unfolded states of the protein are essentially the same, any contribution of the inner filter effect would manifest as a constant baseline effect throughout the experiment, which has been corrected for.

### Analysis of the DSC‐Data

**Figure 5:**
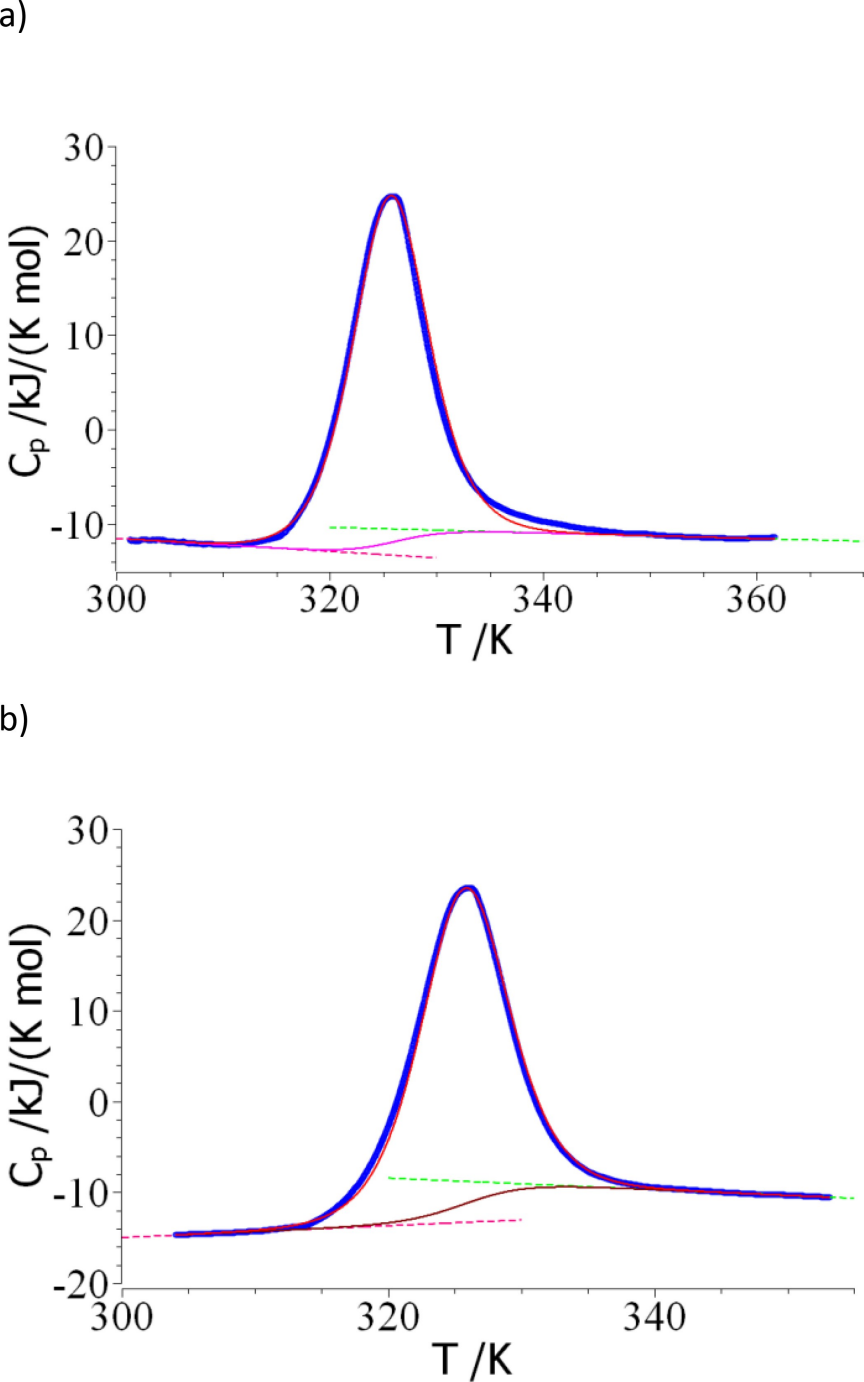
Evaluation of the DSC‐data obtained on a solution of lysozyme at pH 2. a): concentration of lysozyme: 0.964 mg/mL (69 μM); b): 2.418 mg/mL (173 μM). The blue points mark the experimental heat signal *C_p_* whereas the dashed lines display the linear extrapolation *C*_*p,f*_ and *C*_*p,u*_ according to eq. (12) and (13). The pink line shows the calculated background *C_bg_*. The red solid lines display the respective fits according to eq.(20).

For a comparison with the nanoDSF results discussed above, DSC measurements were conducted at *c_protein_* =69 μM (0.964 mg/ml) and 173 μM (2.418 mg/ml)) as shown in Figure 5a and b, respectively. Here the heat capacity below and above the transition is approximated linearly according to eq.(12) and (13). The red and green dashed lines show the fit below (*C*_*p,u*_) and above (*C*_*p,f*_) the transition, respectively. Evidently, the linear approximation leads to a good excellent fit of the data. In principle, this fit could be done together with the fit of the entire *C_p_(T)*.^[59]^ The present procedure, however, ensures that the underlying assumptions and possible errors of the fit can be assessed separately at each stage. The change of the specific heat *Δc_p_* can directly been read off from the difference of the heat capacities at *T_m_*:^[7]^

(23) $$\Delta c_{p}=\;C_{p,u}\left(T_{m}\right)-\;C_{p,f}\left(T_{m}\right)$$

This determination is afflicted by an appreciable error. The values *Δc_p_* =2.78 kJ/(K mol) derived from the data displayed in Figure 5a, and *Δc_p_* =4.76 kJ/(K mol) from the data in Figure 5b, however, are good enough for the subsequent evaluation of the data.

For the evaluation of the DSC‐data we developed an iterative procedure which will be exemplified for the data shown in Figure 5a: For the calculation of *ΔH_cal_* according to eq.(10) $C_{p}^{ex}$ must be evaluated first through eq.(15) which requires the subtraction of the background signal *C_bg_*. The evaluation of this quantity according to eq.(14), however, requires the degree of unfolding *α* which is not available at this stage. A trial value of *ΔH_cal_* can be obtained through subtraction of *C*_*p,f*_ *(T)* from *C_p_* for *T ≤ T_m_*. For *T > T_m_*, *C*_*p,u*_ *(T)* is subtracted from the measured heat capacity *C_p_*. Hence, we obtained trial values for $C_{p}^{ex}$ that yield *ΔH_cal_* =344.5 kJ/mol upon numerical integration. In the next step, *ΔH_u_* is determined by use of eq.(21). $C_{p}^{ex}\left(T_{m}\right)$ can be approximated by the mean value of the trial values of this quantity obtained at step 2. Here we obtain *ΔH_u_* =389.6 kJ/mol and *T_m_* =325.7 K. The latter value is taken as the improved transition temperature and used in all subsequent steps.

Given *T_m_*, *ΔH_u_* and *Δc_p_*, the free energy *ΔG_u_* can be calculated according to eq.(2). This allows us to calculate *C_bg_* according to eq.(14). The pink line in Figures 1 shows this new background line. Subsequently, *C_bg_* can be subtracted from *C_p_* to yield an improved value of *C_p_* ^*ex*^ (eq.(15)). Numerical integration of the improved *C_p_* ^*ex*^ leads to *ΔH_cal_* =344.8 kJ/mol which agrees very well with the value determined in step 2. Hence, *ΔH_cal_* =344.8 kJ/mol is used in all subsequent calculations, no further iteration is done. *C_p_* ^*ex*^ shown in Figure 2 can now be fitted by eq.(20). Here *ΔH_u_* is treated as the only adjustable parameter. The red line in Figure 2 shows the resulting fit which leads to *ΔH_u_* =375.2 kJ/mol. Application of eq.(22) yields 374.9 kJ/mol so that in the following a value *ΔH_u_* =375 kJ/mol is used. Since these data indicate self‐consistency, no further iteration must be done. Table 1 summarizes the results obtained for both protein concentrations.

A comparison of the data gathered in Table 1 shows that the transition temperatures measured by DSC are slightly lower than the ones measured by nanoDSF. Moreover, the enthalpies determined by both methods demonstrate clearly that higher protein concentrations are followed by higher enthalpies of unfolding. This finding is in accord with results of Kitamura and Sturtevant^[47]^ who studied systematically the dependence of thermodynamic parameters of the unfolding of lysozyme of the T4‐bacteriophage on protein concentration (cf. Table 1 of ref.^[47]^). With respect to *Δc_p_* the DSC results show a significant increase with increasing concentration. This is not mirrored in the nanoDSF results. However, as already discussed above, this parameter is afflicted by a considerable error. In particular, its determination based on fits of *F_350_* is sensitive to the quality of the description in the one‐state regions which is worse for the data set at *c_protein_* =100 μM.

It is interesting to note that the ratio of *ΔH_u_/ΔH_cal_* =1.09 obtained from DSC‐measurements at *c_protein_* =69 μM is in a range expected for the two‐state folding model of protein unfolding.[^6^, ^7^, ^8^] A much higher value results for the highest concentration under consideration here (see Table 1). Thus, a value of *ΔH_u_/ΔH_cal_* =1.31 is already outside the range in which the two‐state folding model can be applied safely.^[8]^ Taking all these results together, it becomes obvious that a higher protein concentration leads to an increase of the unfolding enthalpy while the caloric enthalpy *ΔH_cal_* is lowered significantly.

A possible reason for this result may be due to association of lysozyme below the temperature of the transition. Since a long time it is known that lysozyme forms small clusters in the unfolded state in aqueous solution due to a balance of short‐range attractive and long‐range repulsive forces.[^62^, ^63^] A comprehensive review of this work has been given by Stradner and Schurtenberger.^[64]^ Hence, formation of clusters is followed by a higher *ΔH_u_* but a lower caloric enthalpy. In addition to this, the parameter *ΔH_U_/*Δ*H_cal_* deviates from unity. This finding indicates that the unfolding of lysozyme may not be described safely in terms of a two‐state process anymore. Figure 4, on the other hand, clearly indicates that cluster formation plays no significant role anymore above the transition temperature.

The Panta device also measures dynamic light scattering simultaneously which allows to extract the temperature dependence of the hydrodynamic radius *R_H_*. This set of data allows to monitor the overall dimensions during the unfolding transition. Attempts to measure *R_H_* at low concentrations as 10 μM failed. For these conditions the scatter of the data was too high which is typically found if the signal of a weakly scattering sample is obscured by the presence of small amounts of strongly scattering objects such as dust particles. A meaningful signal could only be obtained for a protein concentration of 100 μM. In this concentration range, however, the effect of association may make itself already felt and the analysis of these data must proceed with caution.

**Figure 6:**
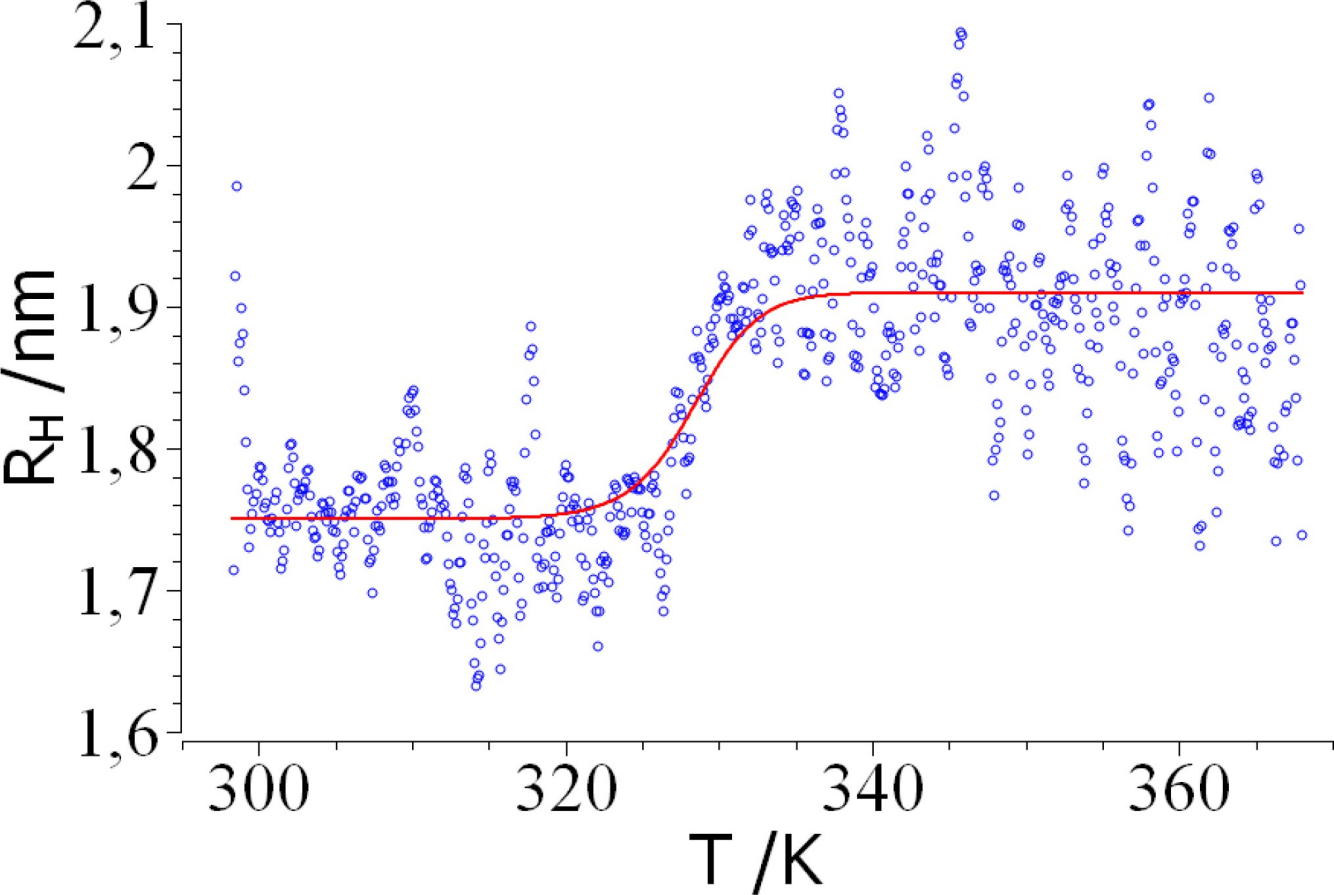
Hydrodynamic radius *R_H_* as the function of temperature measured at a protein concentration of 100 μM. The solid line denotes the fit of the data according to eq.(22).

Figure S5 of the SI displays the raw data that still exhibit a strong scatter of the data. To compare these data with theory, outliers of *R_H_*, that is, values of *R_H_* tenfold larger than the average were removed first. Then a Savitzky‐Golay filtering was applied to the data (2^nd^ order polynomial, ±5 points). The resulting *R_H_* as the function of temperature is shown in Figure 6. The scatter of the data is still quite high but the transition is clearly visible. The solid line shows the fit of these data according to eq.(22) where *R*_*H,f*_ and *R*_*H,u*_ denote the hydrodynamic radius of the folded and the unfolded state of lysozyme, respectively. The degree of unfolding was calculated using eq.(4) using the parameters obtained for a protein concentration of 100 μM (see Table 1). The radii *R*_*H,f*_ and *R*_*H,u*_ have been treated as fit parameters yielding *R*_*H,f*_ =1.75 nm and *R*_*H,u*_=1.91 nm. Full agreement of theory and experiment is seen which further underscores the validity of the two‐state folding model.

The hydrodynamic radii obtained herein compare favorably with the radii of gyration *R_g_* determined by Hirai et al. analyzing small‐angle x‐ray scattering data.^[65]^ These authors studied the unfolding of lysozyme by measuring *R_g_* as the function of temperature and pH. For a pH of 1.2 they found a radius of gyration of ca. 1.6 nm whereas a value of 1.9 nm was deduced for the unfolded state at 80 °C. Kamatari et al. found a radius of gyration in the native state of 1.57 nm in their study, also done by small‐angle X‐ray scattering.^[66]^ Since the hydrodynamic radius for a compact object should be larger than its radius of gyration, full agreement with the present data can be seen for the unfolded state. Unfolding of lysozyme in presence of methanol led to a radius of 2.87 nm as found by Kamatari and coworkers.^[67]^ However, Katamari et al. demonstrated that denaturation by alcohols lead to an extended helical structure which is larger than a globular conformation. The rather small value for the dimensions in the unfolded state of 1.9 nm found here and by Hirai et al.^65^ shows that the unfolded protein assumes a rather dense structure which is in general agreement with early theoretical deductions[^68^, ^69^] and a more recent analysis.^[70]^

### Dependence on pH

In literature thermodynamic properties of the folding/unfolding transition of lysozyme were also deduced from pH‐dependent measurements (see the discussion e. g. in ref.[^47^, ^48^, ^71^, ^72^]). In this way the transition enthalpy was obtained for different temperatures which can be used as an alternative approach to determine the change of *Δc_p_*.^[44]^ Moreover, the dependence of *T_m_* on pH gives information about the number of exchanged protons during the transition. In this way the dependence of *T_m_* and $\Delta H_{u}$ gives highly valuable information on the unfolding transition. It is thus interesting to compare these results with pH‐dependent nanoDSF measurements. The latter were performed at pH 2 and 2.8 using a glycine buffer whereas the pH range 4–6 was adjusted by a citrate buffer. All buffers had a total salt concentration of 50 mM. Based on the discussion presented above (see the discussion of Figure 3 above) a reliable extraction of thermodynamic data was possible up to a pH of 5. The respective intensities *F_350_* measured at different pH are gathered in Figures S6–S10.

**Figure 7:**
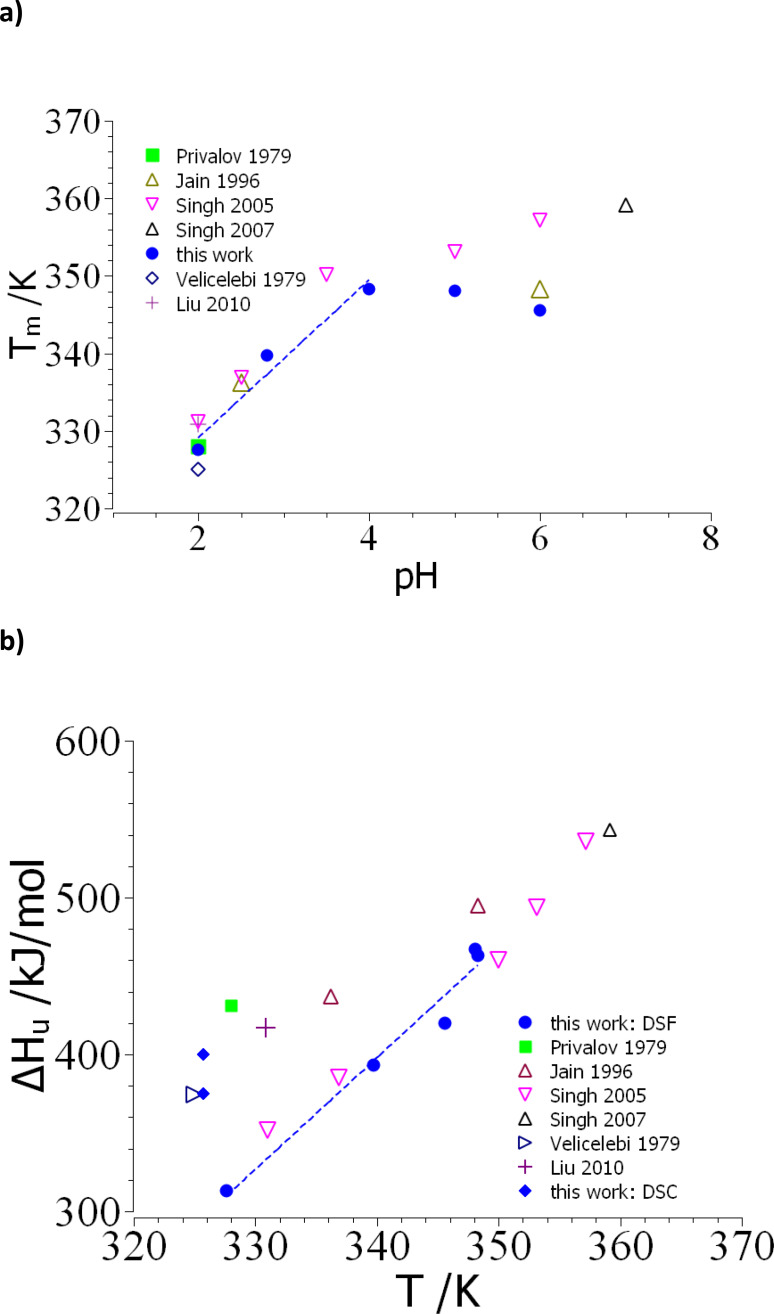
a,b. Survey of thermodynamic data and comparison to literature. Figure 7a) displays the transition temperatures as the function of pH whereas Figure 7b) shows the respective unfolding enthalpies as the function of the unfolding temperature *T_m_*. The filled blue circles refer to the DSF‐data obtained herein whereas the filled blue diamonds refer to the DSC‐data obtained in this work. The other data refer to different techniques: DSC‐data: Privalov 1979;^[1]^ Velicelebi and Sturtevant 1979,^[7]^ Jain and Ahluwalia 1996,^[48]^ Liu et al. 2010.^[43]^ UV‐Vis: Singh et al. 2005^[53]^ and Singh et al. 2007.^[54]^ The dashed line shows the linear fit to the filled blue circles from which *Δc_p_* =7.1 kJ/(K mol) can be derived.

Figure 7a displays the measured transition temperatures as the function of pH whereas Figure 7b shows the respective transition enthalpies as the function of *T_m_*. Table 1 gathers all data derived from this analysis in the present work. The literature data in Figure 7a have been obtained by DSC,[^1^, ^7^, ^43^, ^48^] and by US/VIS‐spectroscopy.[^53^, ^54^] The agreement of data in general is very good at low pH whereas differences are seen beyond a pH of 4. In this region of higher pH‐values, however, the transition is only partially reversible which may impede the entire thermodynamic analysis. In general, the survey shown in Figure 7a suggests that *T_m_* is a rather robust quantity that may be obtained by quite different methods in a secure fashion. This finding is in full agreement with the findings discussed above.

Figure 7a shows that *T_m_* seems to go through a shallow maximum which is located around a pH of 4. Such a maximum of the transition temperature as the function of pH is a very common feature observed for many proteins.^[60]^ Often it is related to the pH of the environment in which the protein or enzyme is working. The dependence of *T_m_* gives direct information on the number of protons *Δν* released or taken up during the unfolding transition according to

(24) $$\Delta \nu =\;\frac{\Delta H_{u}^{0}}{2.303\;RT_{m}^{2}}\frac{dT_{m}}{dpH}\;$$

The dependence of *T_m_* between pH 2–4 is indicated by a dashed line in Figure 7a and leads to a value of *Δν* ∼ 2 for this pH‐range, i. e., two protons are taken up during unfolding. This value agrees approximately with data obtained earlier on different mutants of lysozyme by the Sturtevant group.[^72^, ^73^]

Figure 7b displays the corresponding transition enthalpies obtained from the analysis within the two‐state folding model. Apart from the nanoDSF and DSC results presented above, results from literature are also included. The latter can be grouped in two sets which have been obtained by DSC and spectroscopic methods (UV/Vis or fluorescence). In the former case we have direct measurements of $\Delta H_{u}$ whereas the second set contains data derived from the application of eq.(2). The enthalpies obtained here by fluorescence spectroscopy (filled blue circles) agree quite well with the ones obtained by UV/Vis‐spectroscopy (triangles up and down; ref.[^53^, ^54^]). The data obtained by DSC here agree well with the corresponding DSC‐data from literature.[^1^, ^7^, ^43^, ^48^] Figure 7b demonstrates that data obtained by DSC are in general systematically higher than the ones obtained by spectroscopic methods. This is in full agreement with the DSC‐data obtained here (see the blue diamonds in Figure 7b). As already discussed above, this finding can be traced back to the higher protein concentrations necessary for a typical DSC‐experiment which is followed by the formation of clusters of native lysozyme.

The observation of $\Delta H_{u}$ for different pH has been the classical method to obtain the change of specific heat *Δc_p_*.[^7^, ^58^] The enthalpy of transition raises with the pH of the solution (cf. Figure 7b) and the increase of $\Delta H_{u}$ is traced back to *Δc_p_*. The dashed line in Figure 7b shows the analysis of the enthalpies derived from nanoDSF. A linear fit to $\Delta H_{u}$ as the function of *T_m_* has a slope of 7.1 kJ/(K mol) which agrees quite well with the value for *Δc_p_* =6.5 kJ/(K mol) found by Velicelebi and Sturtevant^[7]^ in the course of DSC‐experiments. It shows that nanoDSF leads to accurate values for the unfolding enthalpy. The value of *Δc_p_* thus derived is considerably smaller, however, than the value found here by analyzing *α(T)* (Table 1). The reason for this obvious discrepancy is not yet clear. It should be kept in mind that the values of $\Delta H_{u}$ plotted in Figure 7b refer to widely different states because they were obtained different pH. A direct determination from the fit of the degree of unfolding *α* for a given pH may thus appear as the more reliable procedure. From this procedure we get a value of *Δc_p_* ~10 kJ/(K mol) which is considerably larger than the value found from Figure 7b. Evidently, *Δc_p_* determined from *α(T)* is afflicted by a large error but the discrepancy seen here seems to be outside of this error. Hence, this problem is certainly worth reconsidering.

## Conclusions

The unfolding transition of lysozyme in aqueous solution has been studied by nanoDSF using the Nanotemper device Panta and by thermal analysis (DSC). For both methods, the degree of unfolding *α* was determined as the function of temperature and evaluated in terms of the classical two‐state folding model. It is shown that the temperature *T_m_* of unfolding can be obtained by nanoDSF with excellent accuracy whereas the unfolding enthalpy $\Delta H_{u}^{0}$ has an error in the range of 5–10 %. The temperatures determined by both nanoDSF and DSC largely agree. A marked dependence of intensity of fluorescence and of $\Delta H_{u}^{0}$ on protein concentration was found by nanoDSF. The increase of the enthalpy of transition with concentration found by nanoDSF could be fully corroborated by DSC and traced back to cluster formation of the unfolded lysozyme. The change of specific heat *Δc_p_* as obtained by DSC and nanoDSF is afflicted by a much larger error. nanoDSF leads to a value of 7.1 kJ/(K mol) deduced from $\Delta H_{u}^{0}$ measured at different pH (see the discussion of Figure 7b) in agreement with literature.^[7]^ The average value 10 kJ/(K mol) found by nanoDSF directly, however, is found to be significantly higher (cf. Table 1). This discrepancy points to a problem of the determination of *Δc_p_* that is in need of further study. The change of the overall size of lysozyme as measured in terms of the hydrodynamic radius *R_H_* was found to be rather small (*R*_*H,f*_ =1.75 nm in the folded state; and *R*_*H,u*_=1.91 nm in the unfolded state). This small change of the overall dimensions of lysozyme during unfolding is in full agreement with data deriving from small‐angle x‐ray scattering.

## Conflict of Interests

The authors declare no conflict of interest.

## Supporting information

As a service to our authors and readers, this journal provides supporting information supplied by the authors. Such materials are peer reviewed and may be re‐organized for online delivery, but are not copy‐edited or typeset. Technical support issues arising from supporting information (other than missing files) should be addressed to the authors.

Supporting Information

## Data Availability

The data that support the findings of this study are available from the corresponding author upon reasonable request.
