# Supplementary material for: Measuring the Thermal Unfolding of Lysozyme: A Critical Comparison of Differential Scanning Fluorimetry and Differential Scanning Calorimetry
Source: ChemistryOpen. 2025 Feb 11;14(6):e202400340. doi: 10.1002/open.202400340 (PMC12138054; doi:10.1002/open.202400340)
Supplement: Supplementary file 1 — Supporting Information [file OPEN-14-e202400340-s001.pdf]

ChemistryOpen

Supporting Information

Measuring the Thermal Unfolding of Lysozyme: A Critical Comparison of Differential Scanning Fluorimetry and Differential Scanning Calorimetry

Weronika Malicka, Yueyue Dai, Andreas Herrmann, Rainer Haag, Matthias Ballauff,*
Marina Pigaleva, Thomas Risse, Daniel Lauster, Iman Asakereh, and Mazdak Khajehpour

Unfolding of Lysozyme as monitored by Differential Scanning Fluorimetry and Thermal Analysis

Weronika Malicka, Yueyue Dai, Andreas Herrmann, Rainer Haag, Matthias Ballauff[*]
Marina Pigaleva, Thomas Risse
Institute of Chemistry and Biochemistry, Freie Universität Berlin, 14195 Berlin, Germany
Daniel Lauster
Institut für Pharmazie, Freie Universität Berlin, 14195 Berlin, Germany
Iman Asakereh, Mazdak Khajehpour
University of Manitoba, Winnipeg, Manitoba R3T 2N2, Canada

SUPPLEMENTARY INFORMATION

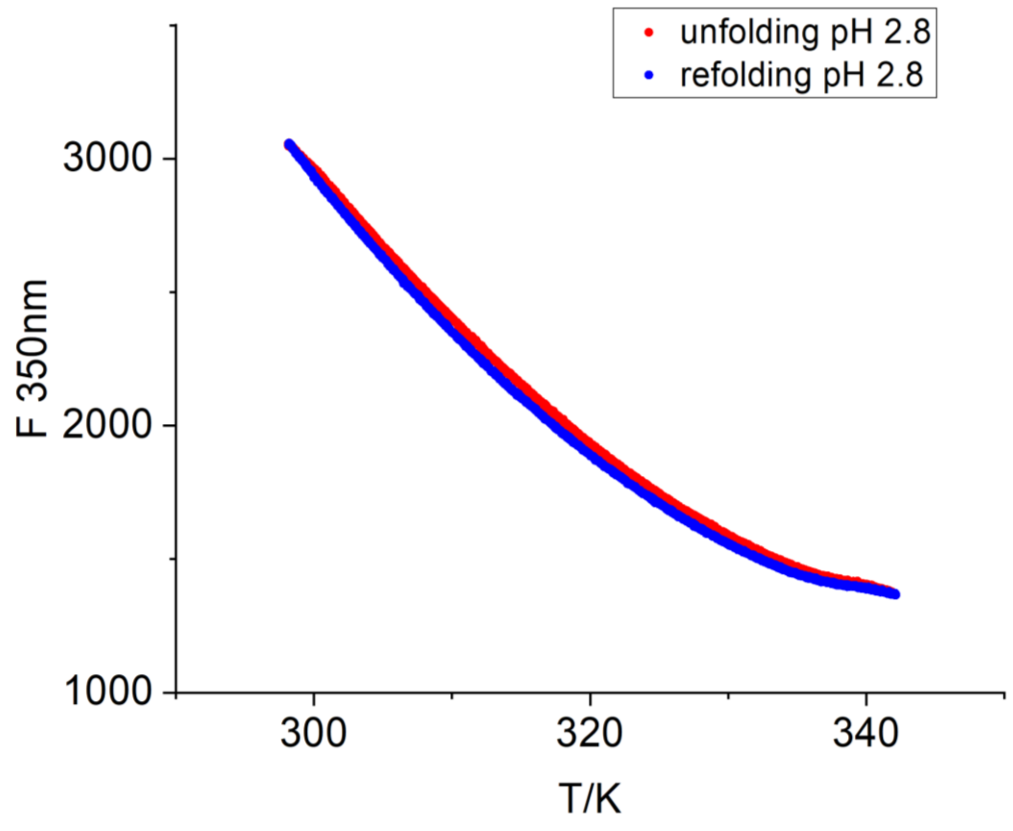

Figure S1. Unfolding (red curve) and refolding (blue curve) of 10 µM lysozyme measured by nanoDSF at 350 nm within the temperature range of 298-342 K at pH 2.8.

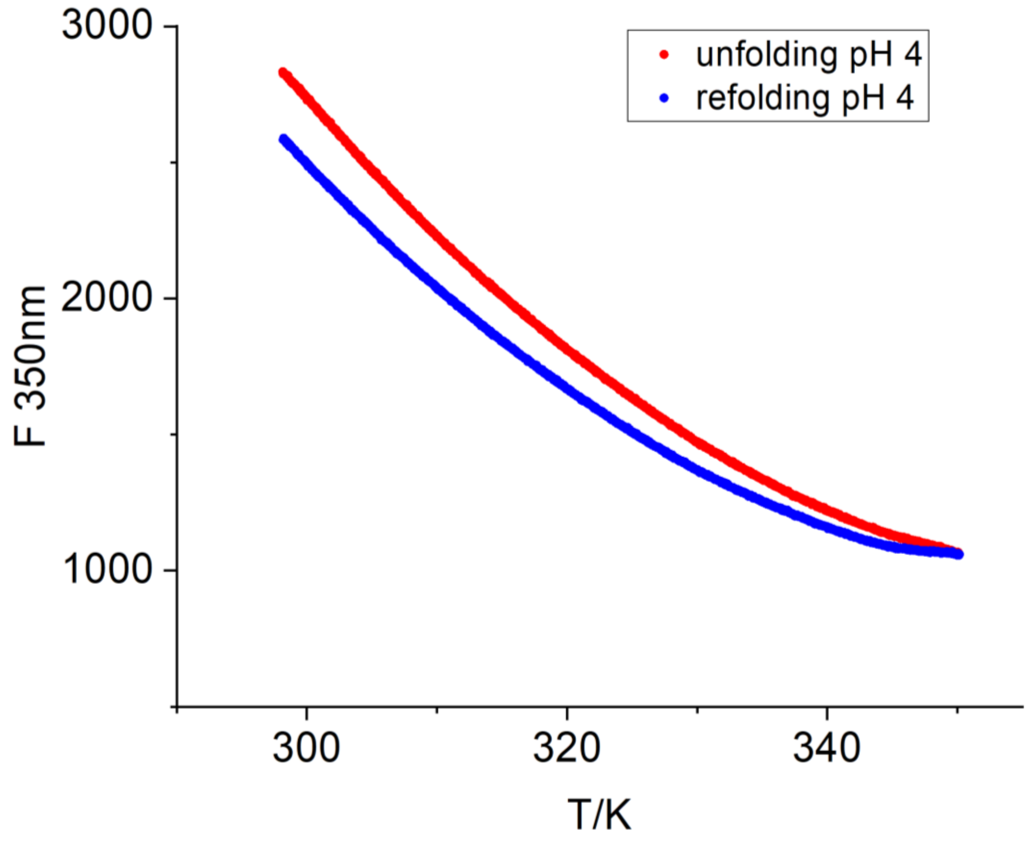

Figure S2. Unfolding (red curve) and refolding (blue curve) of 10 µM lysozyme measured by nanoDSF at 350 nm within the temperature range of 298-350 K at pH 4.

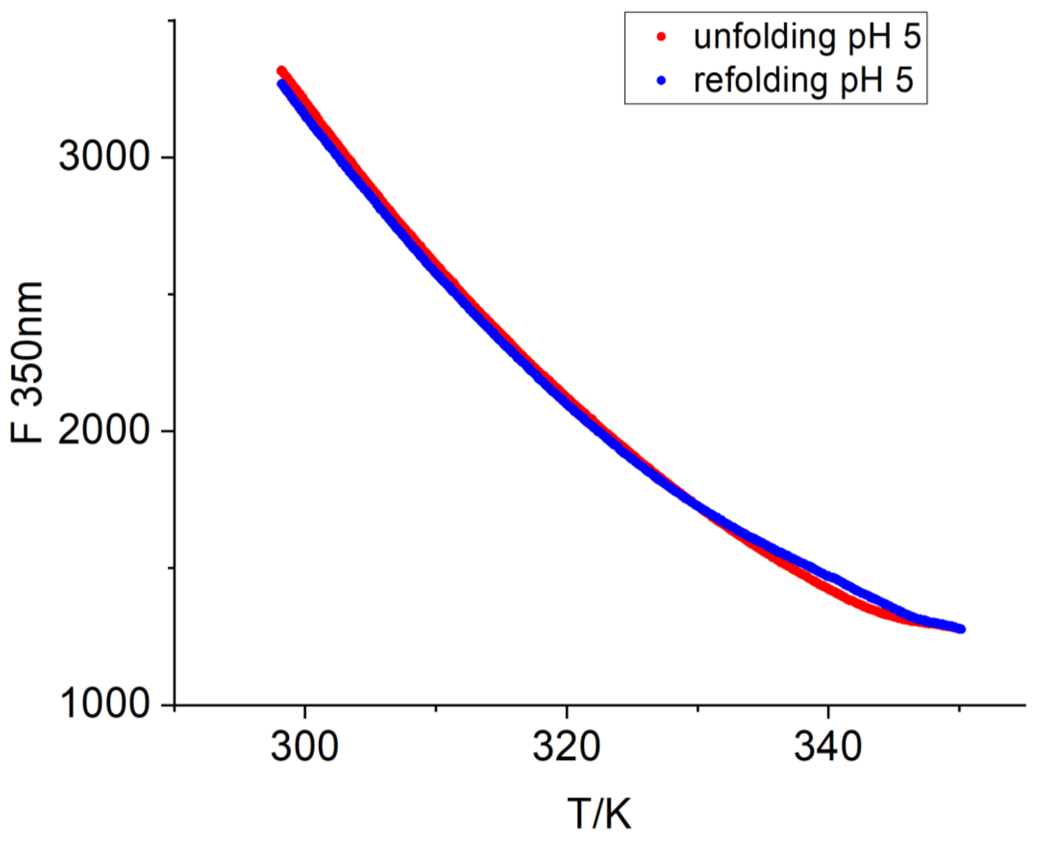

Figure S3. Unfolding (red curve) and refolding (blue curve) of 10 µM lysozyme measured by nanoDSF at 350 nm within the temperature range of 298-350 K at pH 5.

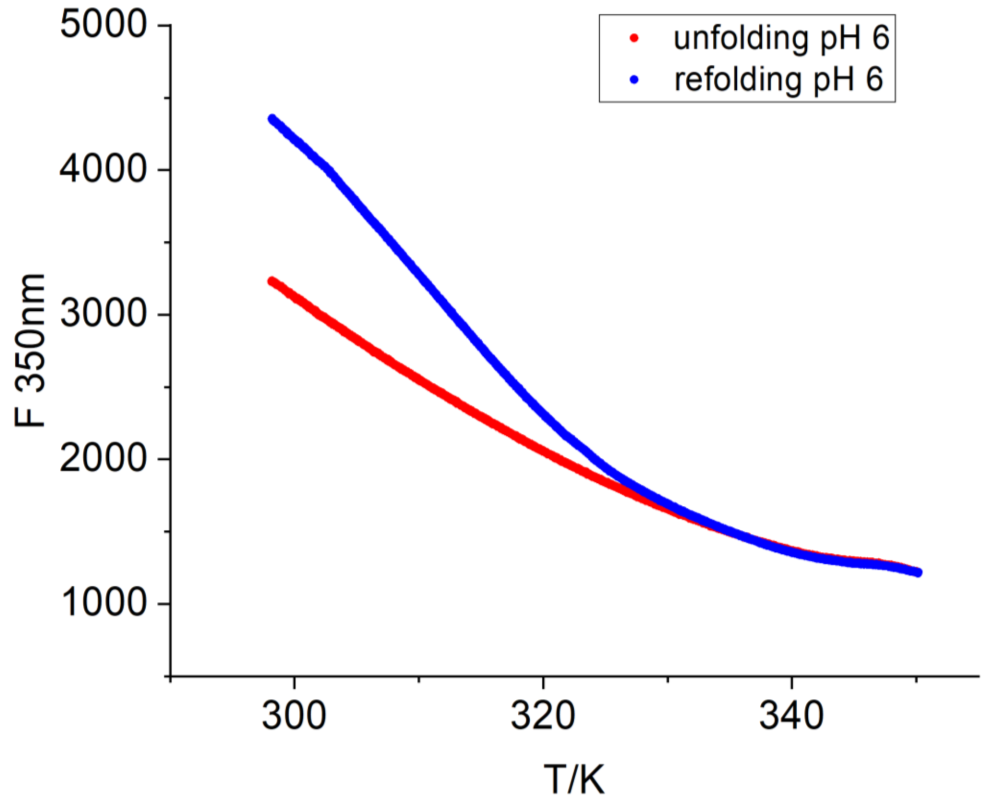

Figure S4. Unfolding (red curve) and refolding (blue curve) of 10 µM lysozyme measured by nanoDSF at 350 nm within the temperature range of 298-350 K at pH 6.

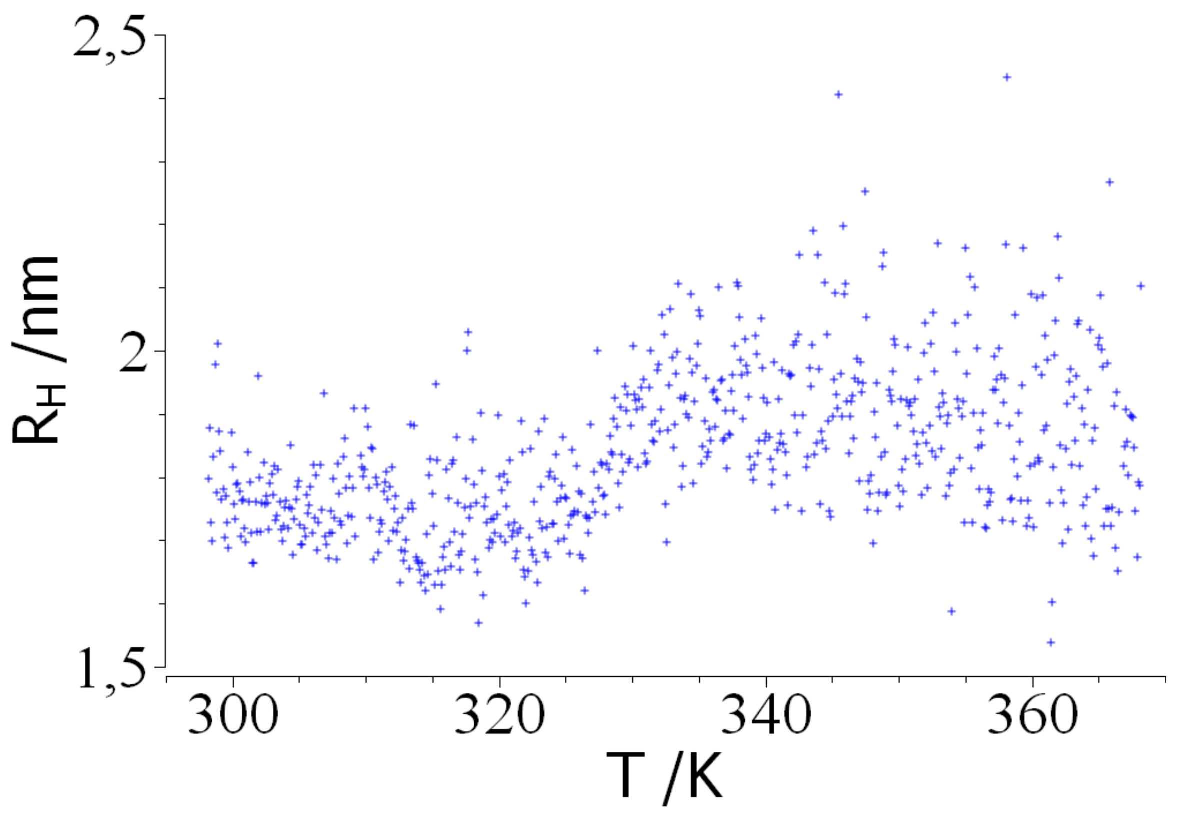

Figure S5. Raw data of the h*ydrodynamic radius R_H as the function of temperature.*

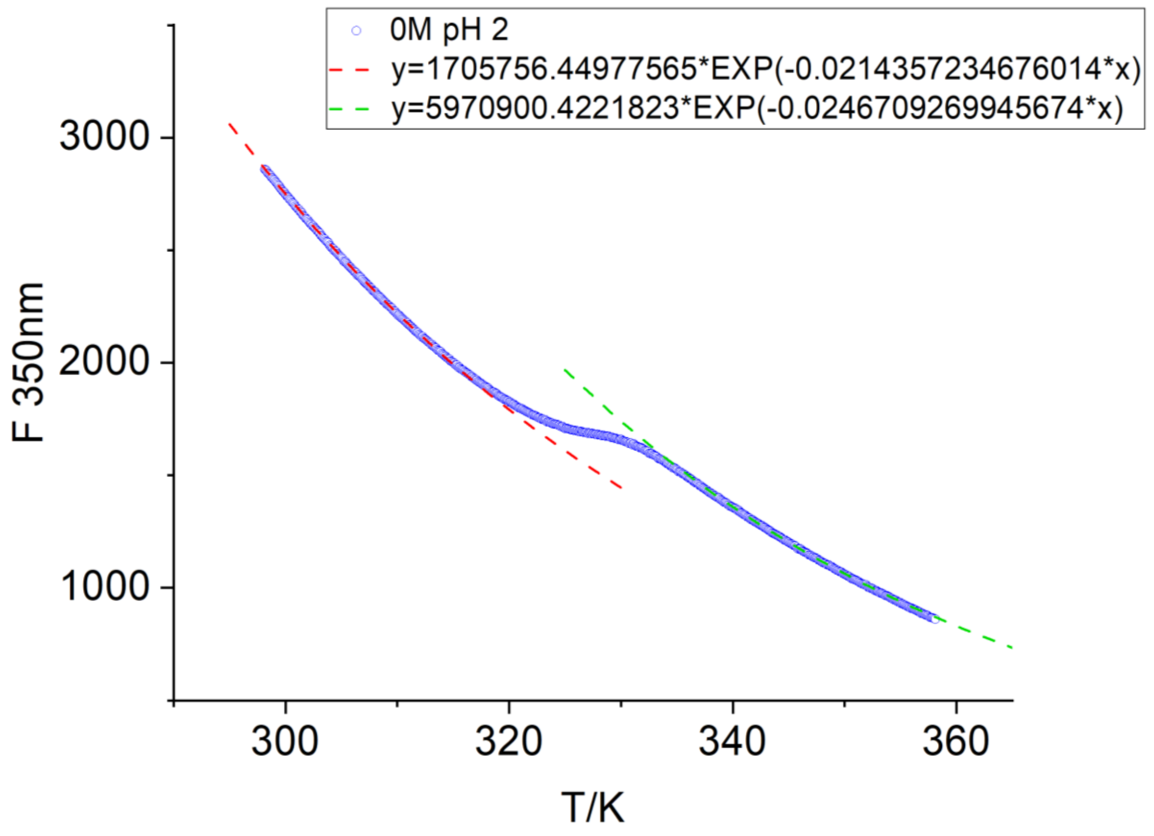

Figure S6. Fluorescence intensities of 10 μM lysozyme in 50 mM glycine buffer (pH 2) without salt measured at 350 nm.The dashed lines display the fits to the unfolded state (red dashed line) and the unfolded state (green dashed line) according to eq.(7).

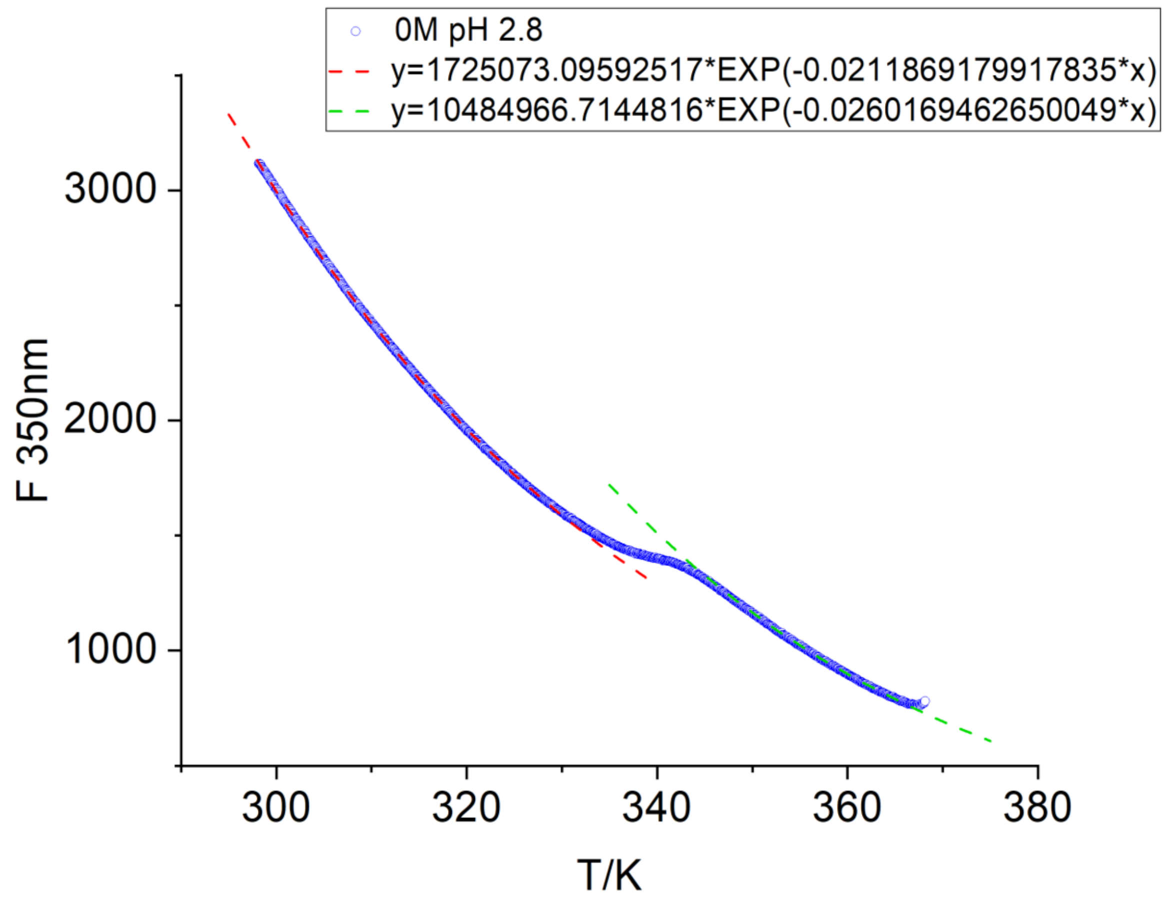

Figure S7. Fluorescence intensities of 10 μM lysozyme in 50 mM glycine buffer (pH 2.8) without salt measured at 350 nm.The dashed lines display the fits to the unfolded state (red dashed line) and the unfolded state (green dashed line) according to eq.(7).

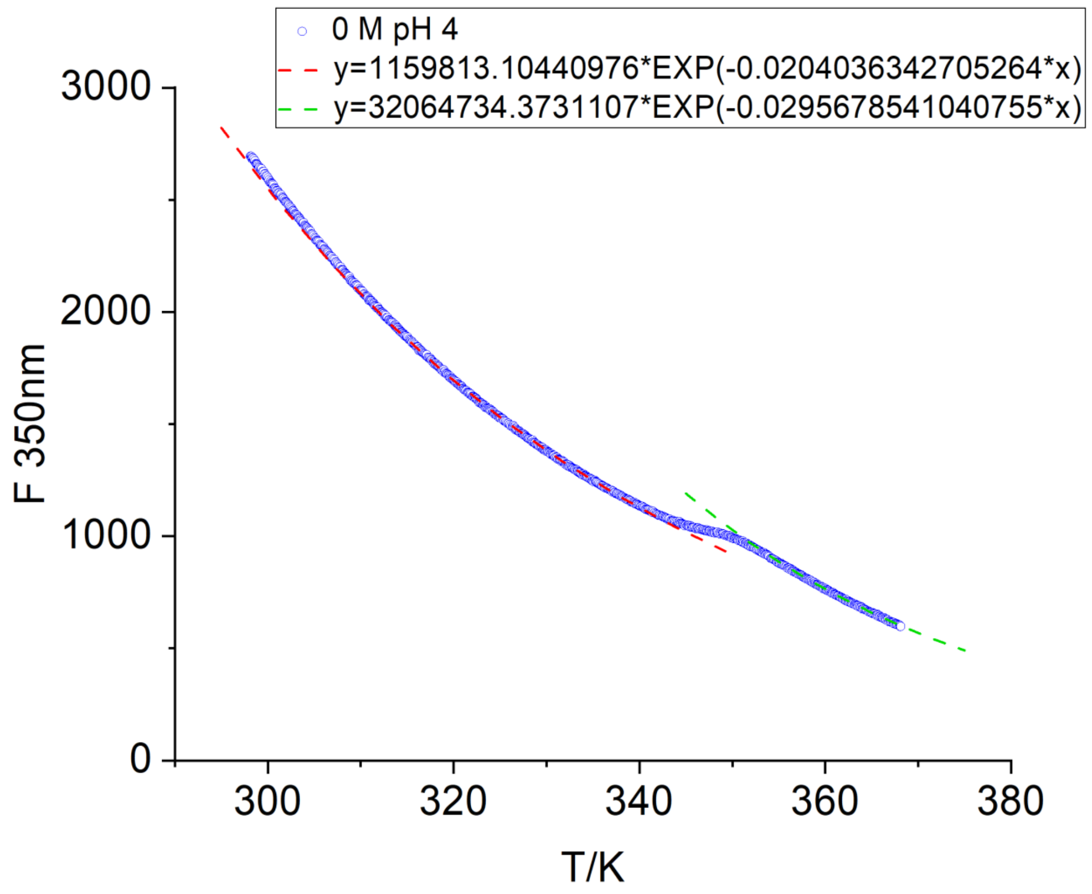

Figure S8. Fluorescence intensities of 10 μM lysozyme in 50 mM glycine buffer (pH 4) without salt measured at 350 nm. The dashed lines display the fits to the unfolded state (red dashed line) and the unfolded state (green dashed line) according to eq.(7).

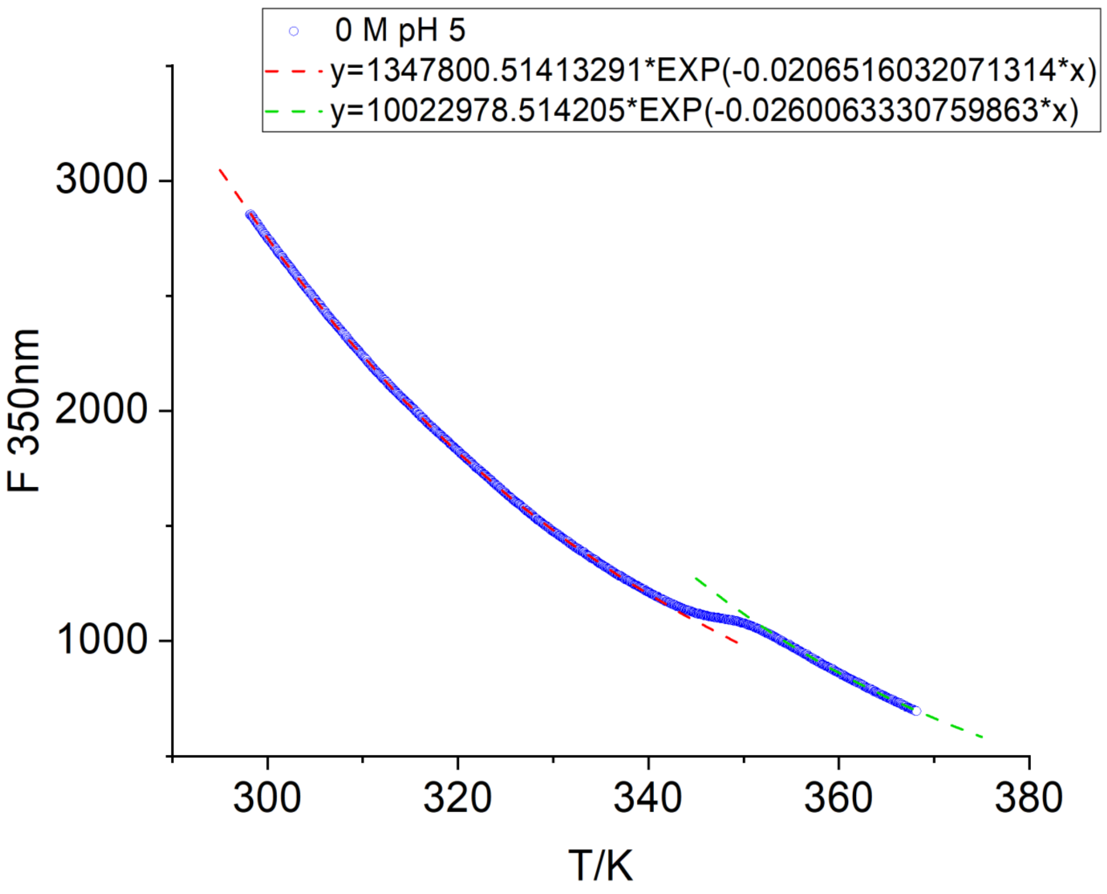

Figure S9. Fluorescence intensities of 10 μM lysozyme in 50 mM glycine buffer (pH 5) without salt measured at 350 nm. The dashed lines display the fits to the unfolded state (red dashed line) and the unfolded state (green dashed line) according to eq.(7).

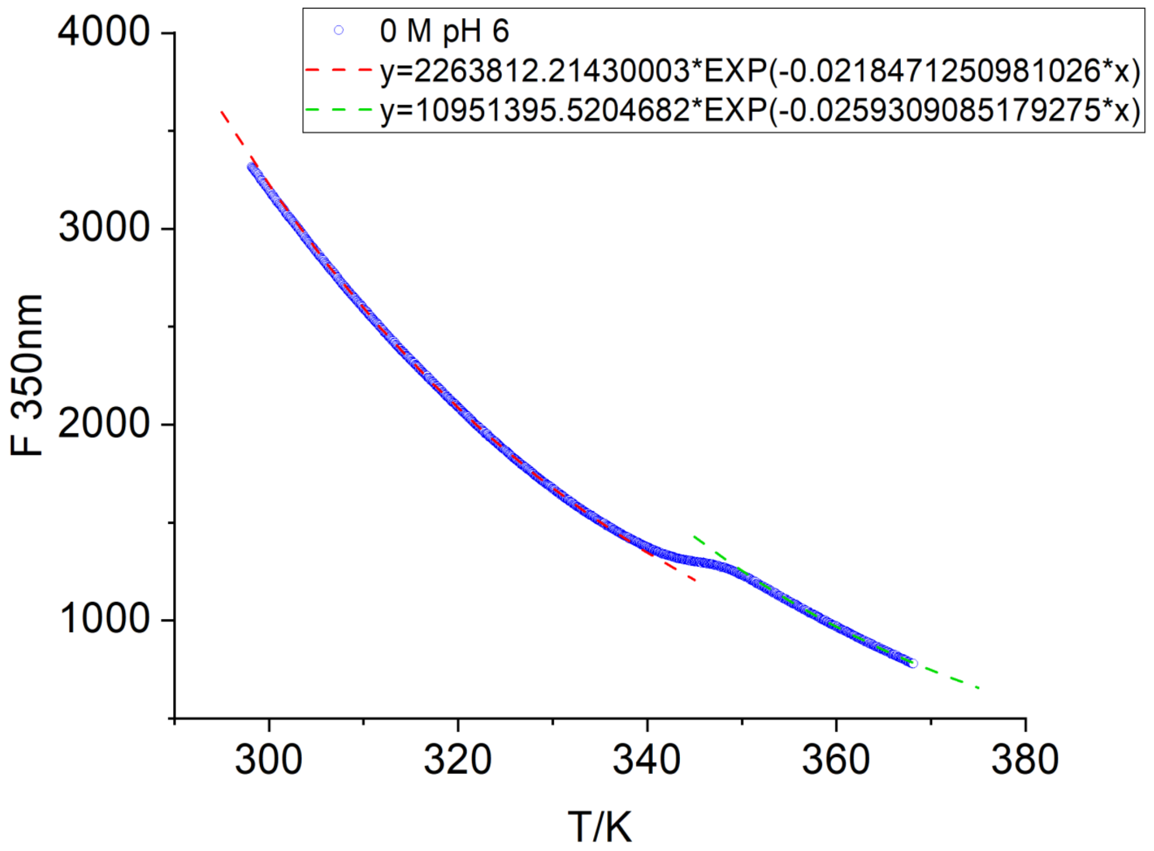

Figure S10. Fluorescence intensities of 10 μM lysozyme in 50 mM glycine buffer (pH 6) without salt measured at 350 nm.The dashed lines display the fits to the unfolded state (red dashed line) and the unfolded state (green dashed line) according to eq.(7).
